# Conservative management of spontaneous pneumothorax: A review of evidence and guidelines

**DOI:** 10.6026/973206300210471

**Published:** 2025-03-31

**Authors:** Yagnang K. Vyas, Jaimin Mansuriya, Krishnakumar Ashokbhai Patel, Yash Bharatkumar Jani, Anushika

**Affiliations:** 1 Department of Respiratory Medicine, Dharmsinh Desai University College Road, Nadiad - 387001, Gujarat, India; 2 Department of Emergency Medicine Dr. N.D. Desai Faculty of Medical Science & Research, Dharmsinh Desai University College Road, Nadiad - 387001, Gujarat, India; 3 Department of Psychiatry Dr. N.D. Desai Faculty of Medical Science & Research, Dharmsinh Desai University College Road, Nadiad - 387001, Gujarat, India

**Keywords:** Spontaneous pneumothorax, primary spontaneous pneumothorax, secondary spontaneous pneumothorax, conservative management, recurrence rates, complications, cost-effectiveness, biomarkers, artificial intelligence, video-assisted thoracoscopic Surgery, pleurodesis, ultrasound, global guidelines

## Abstract

The role of conservative management in spontaneous pneumothorax, an increasingly recognized alternative to invasive procedures is of interest. Recent evidence suggests that observation, oxygen therapy and symptom-based care provide comparable outcomes to invasive methods in stable primary spontaneous pneumothorax. Conservative strategies are associated with reduced complications, shorter hospital stays and improved patient satisfaction. However, controversies exist regarding secondary spontaneous pneumothorax, recurrence risks and the need for standardized guidelines. Further research is needed to optimize conservative approaches and integrate emerging technologies into patient care.

## Background:

Spontaneous pneumothorax is defined as the presence of air within the pleural space, leading to partial or complete lung collapse without external trauma. Spontaneous pneumothorax is classified into primary spontaneous pneumothorax and secondary spontaneous pneumothorax. Primary spontaneous pneumothorax occurs in individuals with no known lung disease, often in tall, thin, young males, typically between 15 and 34 years old. In contrast, secondary spontaneous pneumothorax arises in patients with underlying pulmonary pathology, such as chronic obstructive pulmonary disease (COPD), cystic fibrosis, or interstitial lung disease and it predominantly affects older populations [1, 2]. Epidemiologically, primary spontaneous pneumothorax has an annual incidence of approximately 18-28 per 100,000 males and 1-6 per 100,000 females. Secondary spontaneous pneumothorax, though less frequent, carries a higher morbidity and mortality burden due to the associated comorbidities. Risk factors include smoking, which increases the likelihood of bleb formation, family history and genetic syndromes like Birt-Hogg-Dubé syndrome (Table 1). The pathophysiology of spontaneous pneumothorax involves the rupture of sub pleural blebs or bullae, leading to air leakage into the pleural cavity. In primary spontaneous pneumothorax, subclinical lung abnormalities and changes in intrathoracic pressure are believed to contribute to bleb formation and rupture. The self-resolution process occurs as pleural air is gradually reabsorbed via the pleural capillaries, facilitated by oxygen supplementation [3, 4]. Traditionally, spontaneous pneumothorax has been managed using invasive strategies, including needle aspiration, chest tube drainage and surgical interventions like video-assisted thoracoscopic surgery (VATS) or pleurodesis. While effective, these interventions carry risks of complications such as infection, prolonged air leaks and pain, as well as increased hospital stays and healthcare costs. The emergence of conservative management has shifted clinical paradigms. Approaches such as observation, oxygen therapy and analgesia aim to minimize procedural risks, particularly in stable primary spontaneous pneumothorax [5]. Recent evidence, including randomized controlled trials and meta-analyses, has demonstrated that conservative management is non-inferior to invasive strategies in terms of lung re-expansion and recurrence rates, while improving patient satisfaction and cost-efficiency (Figure 1 - see PDF). Therefore, it is of interest to review on conservative management of spontaneous pneumothorax.

The (Figure 1 - see PDF) provides a structured framework for the management of spontaneous pneumothorax by evaluating patient stability, pneumothorax size and clinical presentation. It begins with the patient presentation, where individuals with suspected spontaneous pneumothorax report symptoms such as sudden chest pain, shortness of breath and reduced breath sounds on the affected side. At this stage, initial imaging is required to confirm the diagnosis. To establish the diagnosis, the primary imaging modality is the chest X-ray, which helps visualize the extent of the pneumothorax and determine its size. In cases where the diagnosis is uncertain, or there are complex presentations, such as secondary spontaneous pneumothorax or small pneumothoraces, a CT scan may be utilized for better precision. Once imaging is complete, the pneumothorax is classified as either small (typically <2 cm from the lung edge to the chest wall) or large and the patient is assessed for clinical stability. Clinical stability is defined as the absence of respiratory distress, hemodynamic instability, or signs of a tension pneumothorax. For patients who are clinically stable and have a small primary spontaneous pneumothorax, the flowchart recommends conservative management. This involves observation and regular monitoring to ensure there is no progression of symptoms.

Additionally, high-flow oxygen supplementation can be provided to accelerate the reabsorption of pleural air, as oxygen reduces the partial pressure of nitrogen in the pleural space. Conservative management is particularly effective in stable cases, as it avoids unnecessary invasive interventions, reduces complications and often allows for outpatient management. In contrast, if the patient is unstable or the pneumothorax is large or secondary, immediate intervention is necessary to restore lung function and prevent further complications. The flowchart outlines options such as needle aspiration, which can be performed to evacuate air in cases of large primary spontaneous pneumothorax, followed by reassessment. Chest tube placement (intercostal drain) is performed to allow continuous air evacuation and lung re-expansion if needle aspiration fails or in unstable secondary spontaneous pneumothorax cases. For patients with persistent air leaks, recurrent pneumothorax, or failure of conservative measures, surgical interventions such as video-assisted thoracoscopic surgery are indicated. Video-assisted thoracoscopic surgery may include pleurectomy or bleb/bullae resection to prevent future recurrences. After intervention or conservative management, follow-up imaging such as chest X-ray or CT scan is conducted to confirm resolution of the pneumothorax and ensure full lung re-expansion. This step is critical in monitoring patient progress and detecting any complications. For cases of recurrent pneumothorax or those with a high risk of recurrence, the flowchart recommends prophylactic measures such as pleurodesis or surgical resection. Pleurodesis achieved chemically or surgically, facilitates the adhesion of the pleural layers, reducing the risk of subsequent pneumothoraces. Surgical resection of blebs or bullae during video-assisted thoracoscopic surgery is also an effective strategy for preventing recurrence, particularly in high-risk patients. In summary, the flowchart emphasizes the importance of evaluating clinical stability and pneumothorax size to guide management decisions. Conservative management, involving observation and oxygen therapy, is prioritized for small, stable primary spontaneous pneumothorax cases to minimize interventions. Conversely, unstable patients or those with large or secondary pneumothorax require immediate intervention through aspiration, chest tube placement, or surgery. Follow-up imaging ensures proper resolution, while prophylactic measures play a key role in managing recurrent pneumothoraces. This structured approach reflects current evidence-based practices, providing a clear and systematic pathway for clinicians to manage spontaneous pneumothorax effectively.

## Historical context of pneumothorax management:

The history of spontaneous pneumothorax management reflects a gradual evolution from invasive interventions to more conservative approaches. Historically, spontaneous pneumothorax was recognized as a medical emergency requiring immediate intervention to prevent life-threatening complications such as tension pneumothorax (Table 2). The uses of chest tube drainage dates back to the early 20th century, following advances in thoracic surgery and understanding of pleural physiology. Initially, large-bore chest tubes were inserted to evacuate pleural air, with prolonged hospital stays being the norm. While effective, this method often resulted in significant morbidity, including infection and patient discomfort [6, 7]. By the mid-20th century, surgical interventions such as thoracotomy and pleurodesis gained prominence, particularly for recurrent spontaneous pneumothorax. Pleurodesis, involving the fusion of pleural layers using chemical agents (*e.g.*, talc) or mechanical abrasion became a standard for preventing recurrence. However, these procedures were invasive, costly and not suitable for all patients, particularly those with primary spontaneous pneumothorax. The advent of video-assisted thoracoscopic surgery in the 1980s revolutionized spontaneous pneumothorax management by providing a minimally invasive alternative to thoracotomy. Video-assisted thoracoscopic surgery allowed for the identification and resection of blebs and bullae, combined with pleurodesis, achieving lower recurrence rates with reduced morbidity. It became the preferred approach for recurrent or persistent spontaneous pneumothorax [8, 9]. Simultaneously, growing evidence suggested that conservative management, including observation and oxygen therapy, could be a safe and effective strategy for stable primary spontaneous pneumothorax. Observation gained traction in the late 20th century as studies demonstrated that pleural air could reabsorb spontaneously without intervention (Table 3). Landmark trials and guidelines, such as the British Thoracic Society (BTS) guidelines, have increasingly supported conservative approaches, particularly for small, stable primary spontaneous pneumothorax. This shift reflects a broader movement toward patient-centered care, balancing the benefits of invasive interventions with the risks of procedural complications. Key milestones in spontaneous pneumothorax research include the recognition of smoking as a major risk factor, advancements in imaging technology such as CT scans and recent randomized controlled trials that validated the safety and efficacy of conservative management [10].

## Pathophysiology and natural course of spontaneous pneumothorax:

The pathophysiology of spontaneous pneumothorax centers on the accumulation of air within the pleural space, disrupting the negative intra-pleural pressure required for lung inflation. This occurs due to the rupture of sub-pleural blebs or bullae, which are small, air-filled sacs that develop on the lung surface (Figure 2 - see PDF) Table 4. In primary spontaneous pneumothorax, the exact mechanisms of bleb formation remain unclear but are believed to involve subclinical lung abnormalities, genetic predispositions and smoking-induced inflammation. Changes in intra-thoracic pressure, such as those induced by coughing, sneezing or physical strain, can lead to bleb rupture, allowing alveolar air to escape into the pleural space [11, 12]. In secondary spontaneous pneumothorax, underlying lung diseases such as chronic obstructive pulmonary disease, interstitial lung disease, cavitary lung disease or cystic fibrosis weaken lung parenchyma, predisposing to air leaks. These conditions lead to poor lung compliance, reduced pleural healing and an increased risk of prolonged air leaks. The natural course of spontaneous pneumothorax involves gradual reabsorption of pleural air. This occurs as nitrogen diffuses from the pleural space into the pleural capillaries. High-flow oxygen therapy accelerates this process by reducing the partial pressure of nitrogen, increasing the diffusion gradient. Several risk factors influence the recurrence and severity of spontaneous pneumothorax. Smoking is the most significant modifiable risk factor, as it induces inflammation and promotes bleb formation. Genetic syndromes, such as Birt-Hogg-Dubé syndrome, increase susceptibility to recurrent spontaneous pneumothorax due to structural lung abnormalities. Other risk factors include male sex, tall and thin body habitus and family history. The process of pleural healing varies between primary spontaneous pneumothorax and secondary spontaneous pneumothorax. In primary spontaneous pneumothorax, pleural defects often heal spontaneously, facilitating lung re-expansion without intervention. In secondary spontaneous pneumothorax, delayed healing due to underlying lung pathology increases the risk of complications such as persistent air leaks and recurrence [13, 14]. Understanding the natural resolution process of spontaneous pneumothorax has underpinned the growing acceptance of conservative management for stable primary spontaneous pneumothorax, as pleural air can reabsorb without the need for invasive interventions [15]. Based upon our experience with Stable secondary spontaneous pneumothorax, conservative treatment is equally effective and it can be done on OPD basis with proper counselling and observation.

## Overview of conservative management approaches:

Conservative management of spontaneous pneumothorax represents a significant shift in clinical practice, offering a non-invasive alternative to traditional surgical and procedural approaches. It involves patient observation, oxygen therapy and symptomatic management to facilitate natural lung re-expansion without immediate intervention [16, 17]. This approach is particularly beneficial in stable primary spontaneous pneumothorax (PSP) cases and is supported by emerging evidence that highlights its safety, efficacy and cost-effectiveness (Table 5). We used conservative approach in mildly symptomatic but stable secondary spontaneous pneumothorax patients who gave negative consent due to procedure related discomfort and unaffordability of cost related to ICD and hospitalization. In addition to above measures for treatment, we also advise to avoid straining or strenuous activity, stop smoking or avoid exposure to smoke, use antitussive drugs, antiemetics, laxatives, analgesics and bronchodilators if breathlessness is on exertion only.

## Definition and components of conservative management:

## The core components of conservative management include:

[1] Observation: Observation involves clinical monitoring of patients without performing invasive procedures. For small pneumothoraces (<2 cm on chest X-ray) in stable primary spontaneous pneumothorax patients, the air in the pleural cavity can be spontaneously reabsorbed over time. Regular follow-up with chest X-rays ensures the pneumothorax does not progress. Observation is typically accompanied by patient education on symptoms of pneumothorax progression, such as increasing breathlessness or chest pain, which may necessitate intervention.

[2] Oxygen therapy: High-flow oxygen therapy accelerates the reabsorption of pleural air by creating a pressure gradient. Oxygen reduces the partial pressure of nitrogen in alveolar gas, enhancing the diffusion gradient for nitrogen to move from the pleural cavity into the capillaries. Studies have shown that oxygen therapy can quadruple the rate of air reabsorption, leading to faster resolution in small pneumothoraces.

[3] Analgesia: Pain management is an essential component of conservative management, as pleuritic chest pain is common in spontaneous pneumothorax. Non-steroidal anti-inflammatory drugs (NSAIDs), such as ibuprofen and acetaminophen are often sufficient. Adequate pain relief improves respiratory mechanics, patient comfort and compliance with conservative management protocols [18].

## Role of imaging in conservative management:

Imaging plays a critical role in confirming the diagnosis of spontaneous pneumothorax, assessing its size, monitoring progression and evaluating lung re-expansion during conservative management.

[1] Chest X-Ray (CXR): Chest X-rays remain the gold standard for diagnosing and monitoring spontaneous pneumothorax. They help determine pneumothorax size, guide decision-making and track lung re-expansion. Serial CXRs are often performed during follow-up visits to confirm resolution.

[2] Computed tomography (CT): While not routinely used in conservative management, CT is invaluable in complex cases where diagnostic uncertainty exists. CT scans can identify small pneumothoraces, blebs and bullae, as well as underlying lung pathology in secondary spontaneous pneumothorax (SSP).

[3] Ultrasound: Point-of-care ultrasound (POCUS) has emerged as a promising, radiation-free alternative for diagnosing and monitoring spontaneous pneumothorax. It is particularly useful in outpatient settings and for pregnant patients where radiation exposure should be minimized. Ultrasound can detect pleural sliding abnormalities, lung points and air accumulation, aiding in bedside assessment [19].

## Evidence supporting the efficacy of conservative approaches:

The adoption of conservative management is underpinned by robust evidence from randomized controlled trials (RCTs), cohort studies and meta-analyses:

[1] Randomized controlled trials (RCTs): Landmark studies, such as Brown *et al.* (2020), demonstrated that conservative management is non-inferior to invasive interventions like needle aspiration or chest tube drainage for stable primary spontaneous pneumothorax patients. At 8 weeks, lung re-expansion rates were comparable between the conservative and interventional groups, with fewer adverse events and complications observed in the conservative group.

[2] Meta-analyses: Systematic reviews and meta-analyses comparing conservative and invasive management have consistently highlighted the advantages of conservative approaches in reducing hospital stays, procedural risks and healthcare costs. Importantly, recurrence rates were found to be similar between the two strategies in stable primary spontaneous pneumothorax patients.

[3] Patient-centered outcomes: Conservative management aligns with patient preferences for non-invasive care. It minimizes hospitalization, reduces procedural anxiety and improves overall quality of life. Patients report higher satisfaction with conservative management due to the avoidance of chest tube placement and associated discomfort [20].

## Advantages and limitations of conservative management:

## Advantages:

[1] Avoids invasive procedures and associated complications.

[2] Reduces hospital admissions and healthcare costs.

[3] Improves patient satisfaction and quality of life.

[4] Suitable for outpatient care with appropriate follow-up.

## Limitations:

[1] Requires close monitoring to detect progression or complications.

[2] Not suitable for unstable or symptomatic patients.

[3] Higher risk of delayed intervention if symptoms worsen.

## Current guidelines and recommendations:

Management of spontaneous pneumothorax is guided by international recommendations, including those from the British Thoracic Society (BTS), the American College of Chest Physicians (ACCP) and the European Respiratory Society (ERS). While these guidelines share common principles, there are notable divergences in specific recommendations regarding conservative management, interventional thresholds and follow-up practices (Table 6) [21, 22].

## Comparative analysis of international guidelines:

## American college of chest physicians (ACCP) consensus (2001):

[1] The American College of Chest Physicians guidelines adopt a more interventional approach compared to other guidelines.

[2] Needle aspiration or chest tube drainage is often preferred, even for small primary spontaneous pneumothorax cases, reflecting a lower threshold for intervention.

[3] Early chest tube placement is strongly recommended for secondary spontaneous pneumothorax due to underlying lung disease and increased mortality risk.

## British thoracic society (BTS) guidelines (2010):

[1] The 2010 BTS guidelines advocate a patient-cantered approach and emphasize the importance of patient stability and pneumothorax size in decision-making.

[2] For small, stable primary spontaneous pneumothorax, observation and oxygen therapy are recommended as first-line treatment.

[3] Needle aspiration is preferred for large primary spontaneous pneumothorax (>2 cm) or symptomatic cases, with chest tube insertion reserved for aspiration failure.

[4] In secondary spontaneous pneumothorax, immediate intervention (*e.g.*, chest tube) is recommended due to the higher risk of complications.

## British thoracic society (BTS) guidelines (2023):

[1] The updated 2023 BTS guidelines maintain the focus on patient-centered care while introducing nuanced recommendations.

[2] Conservative management, including observation and oxygen therapy, remains the first-line approach for small, stable primary spontaneous pneumothorax, in alignment with prior recommendations.

[3] The 2023 guidelines emphasize the utility of point-of-care ultrasound (POCUS) for diagnosis and monitoring, offering a radiation-free alternative.

[4] Needle aspiration remains the preferred method for symptomatic or large primary spontaneous pneumothorax (>2 cm), with chest tube insertion recommended for aspiration failure or unstable patients.

[5] Ambulatory management, using portable devices such as Heimlich valves, is now explicitly encouraged for stable secondary spontaneous pneumothorax and large primary spontaneous pneumothorax to reduce hospitalization.

[6] Surgical interventions, such as video-assisted thoracoscopic surgery with pleurodesis, are highlighted for recurrent pneumothorax or cases of persistent air leak.

## European respiratory society (ERS) guidelines (2023):

[1] The ERS guidelines align closely with the BTS in supporting conservative management for small, stable primary spontaneous pneumothorax cases.

[2] ERS highlights the role of ambulatory management using portable drainage devices, which facilitate outpatient care and reduce hospitalization.

[3] Advanced imaging, such as CT, is recommended for complex or recurrent cases to guide intervention.

## Consensus on indications for conservative management:

International guidelines have increasingly recognized conservative management as an appropriate first-line approach for specific patients with primary spontaneous pneumothorax (PSP). The criteria for conservative management include:

[1] Small pneumothorax (<2 cm): Patients with minimal pleural air on chest imaging.

[2] Clinical stability: Absence of respiratory distress, hemodynamic instability, or signs of tension pneumothorax.

[3] Minimal symptoms: Patients with mild or no dyspnea and minimal pain.

The BTS and ERS guidelines strongly recommend conservative management in such cases, emphasizing the role of close clinical monitoring and serial imaging to ensure safety. In contrast, the American College of Chest Physicians recommends a lower threshold for intervention, often advocating needle aspiration even in small primary spontaneous pneumothorax cases. This discrepancy reflects a more cautious approach in certain clinical settings, particularly in resource-rich environments [23].

## Divergences in recommendations and their implications:

While there is general agreement on conservative management for small, stable primary spontaneous pneumothorax, significant variations exist regarding several aspects of clinical practice:

[1] Threshold for intervention:

a. The BTS and ERS advocate for observation in clinically stable patients with small pneumothoraces, emphasizing non-invasive management to minimize complications.

b. The American College of Chest Physicians, however, recommends immediate intervention (needle aspiration) for most cases, even for small primary spontaneous pneumothorax, reflecting a lower threshold for invasive treatment.

[2] Implication: These differing recommendations result in variability in clinical practice. In settings influenced by the American College of Chest Physicians, patients may undergo invasive interventions unnecessarily, whereas BTS- or ERS-guided care may prioritize observation, avoiding procedural risks. This underscores the need for globally unified guidelines to balance conservative and interventional approaches effectively.

[3] Management of secondary spontaneous pneumothorax (SSP):

a. Guidelines uniformly recommend immediate intervention for secondary spontaneous pneumothorax due to its higher risk of respiratory compromise. Chest tube insertion remains the preferred option in unstable or symptomatic SSP.

b. Conservative management is not routinely advocated for secondary spontaneous pneumothorax but may be considered in exceptional circumstances for stable, minimally symptomatic patients, typically with close monitoring [24].

[4] Implication: The emphasis on early intervention for secondary spontaneous pneumothorax reflects the severity of the condition. However, the lack of evidence supporting conservative management in specific, stable secondary spontaneous pneumothorax cases limits options for patient-centered care. Future research should explore whether selected secondary spontaneous pneumothorax patients might benefit from conservative approaches, potentially broadening management strategies for this subgroup.

[5] Role of ambulatory management devices:

a. The ERS strongly supports the use of ambulatory devices, such as Heimlich valves, enabling outpatient management for large primary spontaneous pneumothorax. These devices have shown potential to reduce hospitalization and improve patient comfort.

b. The BTS and American College of Chest Physicians provide limited recommendations on ambulatory devices, reflecting slower adoption and varying clinical practice preferences across regions.

[6] Implication: The divergence in the adoption of ambulatory devices highlights the need for additional research to validate their safety, efficacy and cost-effectiveness across diverse populations. Wider acceptance of these devices could lead to substantial improvements in resource utilization and patient satisfaction, particularly in stable cases.

[7] Follow-Up practices:

a. The BTS recommends serial chest X-rays during conservative management to monitor resolution and detect complications.

b. The ERS goes further by promoting advanced imaging techniques, such as CT, particularly for recurrent or complex cases where underlying pathology must be assessed.

[8] Implication: Variability in follow-up protocols can affect both healthcare resource use and patient outcomes. In settings with limited access to advanced imaging, reliance on chest X-rays may delay the identification of underlying issues. Standardized follow-up practices, tailored to resource availability, would enhance care delivery and patient safety.

## Discussion of guideline updates and controversies:

Recent updates to international guidelines reflect the growing body of evidence supporting conservative management for primary spontaneous pneumothorax. Key developments include:

[1] Validation of conservative management: The results of landmark trials, such as Brown *et al.* (2020), have strongly influenced the BTS and ERS guidelines, leading to greater emphasis on observation and oxygen therapy for small, stable primary spontaneous pneumothorax. However, the American College of Chest Physicians has yet to fully integrate these findings, maintaining a preference for early intervention.

[2] Ambulatory management controversy: Ambulatory devices, such as Heimlich valves and portable pleural drains, offer promising alternatives to traditional chest tube placement. While the ERS guidelines actively promote their use, concerns remain regarding patient selection, follow-up protocols and complication rates. Additional randomized controlled trials are needed to establish their role in routine practice.

[3] Management of recurrent pneumothorax: Surgical interventions, such as video-assisted thoracoscopic surgery (VATS) with pleurodesis, remain the gold standard for recurrent or persistent spontaneous pneumothorax. However, the role of conservative management in reducing recurrence remains a topic of on-going research.

[4] Standardization of definitions: One of the persistent challenges in spontaneous pneumothorax management is the lack of standardized definitions for key terms, such as "small pneumothorax" and "clinical stability." This inconsistency can lead to variations in patient selection and treatment strategies across different guidelines [25].

## Evidence from clinical trials and studies:

The growing acceptance of conservative management for spontaneous pneumothorax (SP) is underpinned by robust evidence from randomized controlled trials (RCTs), cohort studies and systematic reviews. This section summarizes key studies, including their design, population, interventions, outcomes and implications for clinical practice (Table 7) [26, 27, 28, 29, 30, 31-32].

## Comparative outcomes: conservative vs. invasive management:

The comparison between conservative and invasive management of spontaneous pneumothorax (SP) has been an area of significant clinical interest. This section evaluates outcomes such as recurrence rates, complications, mortality, hospitalization duration, cost-effectiveness and quality of life to provide a comprehensive understanding of the advantages and limitations of each approach (Table 8) [33, 34, 35, 36-37].

## Recurrence rates, complications and mortality:

[1] Recurrence rates: Recurrence remains a major concern in the management of spontaneous pneumothorax, especially in patients managed conservatively. Studies have demonstrated that while conservative management is safe and effective in the short term, recurrence rates can be slightly higher when compared to surgical interventions. For instance, observational studies and meta-analyses show recurrence rates between 15-30% for conservative approaches. In contrast, surgical interventions such as video-assisted thoracoscopic surgery achieve recurrence rates of less than 5%, particularly when combined with pleurodesis.

a. The key factor contributing to recurrence in conservative management is the absence of pleural intervention. While observation allows the lung to re-expand naturally, it does not address underlying issues such as blebs or bullae, which may rupture again. Surgical approaches, on the other hand, proactively address these issues through resection and pleurodesis, reducing the likelihood of recurrence.

[2] Complications: Complications are significantly reduced with conservative management compared to invasive approaches. Conservative strategies avoid risks such as infections, prolonged air leaks, bleeding and pain associated with chest tube insertion or surgical interventions. Clinical trials, such as the Brown *et al.* (2020) study, have reported adverse events of 4.1% in conservative groups compared to 16.8% in interventional groups. This difference highlights the procedural risks associated with invasive methods.

[3] Mortality: Mortality rates for primary spontaneous pneumothorax (PSP) are extremely low, regardless of the management strategy, given the typically healthy baseline status of affected individuals. However, in secondary spontaneous pneumothorax (SSP), mortality is a greater concern due to underlying lung diseases such as chronic obstructive pulmonary disease or interstitial lung disease. Conservative management may not be appropriate in secondary spontaneous pneumothorax, as delayed intervention could worsen respiratory failure. Mortality in secondary spontaneous pneumothorax is often related to comorbidities rather than pneumothorax itself [38, 39-40].

## Hospitalization duration and cost-effectiveness:

[1] Hospitalization duration: Conservative management has been shown to significantly reduce hospitalization duration, as many patients can be managed on an outpatient basis with careful monitoring. In stable primary spontaneous pneumothorax, patients observed conservatively often require only brief follow-up appointments with chest X-rays, allowing them to recover at home. In contrast, invasive management, such as chest tube insertion, necessitates hospitalization for continuous monitoring of air drainage and lung re-expansion [41, 42- 43].

a. Ambulatory management devices, such as Heimlich valves and portable pleural drains, further enhance the feasibility of outpatient care. Studies have reported hospital stays of 0-2 days for conservative management compared to 3-7 days for invasive approaches.

[2] Cost-Effectiveness: Conservative management offers significant cost savings compared to invasive methods. By avoiding hospitalization, procedural costs and potential complications, conservative management reduces the overall financial burden on healthcare systems. Economic analyses have shown that the cost of observation and oxygen therapy is a fraction of the cost associated with chest tube insertion, video-assisted thoracoscopic surgery or pleurodesis.

a. Furthermore, outpatient-based ambulatory management further improves cost-effectiveness while maintaining safety and efficacy [44, 45-46].

## Quality of life and patient satisfaction:

[1] Quality of life: Quality of life (QoL) outcomes favour conservative management due to its non-invasive nature and reduced physical and emotional burden. Patients managed conservatively report less pain, reduced anxiety and faster return to daily activities. Avoidance of invasive procedures such as chest tube placement also improves patient comfort during recovery. In contrast, while surgical interventions achieve lower recurrence rates, they are associated with higher short-term morbidity, including post-operative pain, scarring and emotional stress. Studies evaluating patient-reported outcomes indicate that patients often prioritize non-invasive options when the risk of recurrence is minimal [47, 48].

[2] Patient satisfaction: Conservative management aligns with patient preferences for less invasive care, particularly in stable primary spontaneous pneumothorax. The ability to avoid hospitalization and resume normal activities quickly contributes to higher patient satisfaction. However, in cases of recurrent spontaneous pneumothorax or prolonged symptoms, patients may prefer definitive surgical management to prevent further episodes. Overall, shared decision-making between clinicians and patients remains essential to balance the benefits and risks of conservative versus invasive management, taking into account individual circumstances and preferences [49-50].

## Subgroup analysis:

## Primary vs. secondary pneumothorax and other risk factors:

[1] Primary spontaneous pneumothorax (PSP): Conservative management is most suitable for stable primary spontaneous pneumothorax patients with small pneumothoraces and minimal symptoms. Evidence supports that primary spontaneous pneumothorax can resolve naturally through air reabsorption, with outcomes comparable to invasive methods. Recurrence rates remain acceptable in most cases, particularly when patients are monitored closely [51, 52- 53].

[2] Secondary spontaneous pneumothorax (SSP): SSP presents additional challenges due to the presence of underlying lung disease, which compromises pulmonary function and increases the risk of complications. Conservative management is rarely recommended for secondary spontaneous pneumothorax due to the higher likelihood of prolonged air leaks and respiratory compromise. Chest tube insertion or surgical interventions remain the preferred options for secondary spontaneous pneumothorax, with close monitoring to prevent deterioration.

[3] Other Risk factors: Smoking: Smoking significantly increases the risk of recurrence, regardless of the management strategy. Smoking cessation is strongly recommended to reduce recurrence rates.

Age and Comorbidities: Older patients and those with comorbidities may have limited pulmonary reserve, making invasive interventions riskier. Conservative management may be preferred in such cases if the patient is stable and closely monitored.

## Pregnancy:

In pregnant patients, conservative management is favored to minimize procedural risks and radiation exposure. Ultrasound is often used for diagnosis and monitoring in this population.

## Challenges and controversies:

Despite the growing evidence supporting conservative management of spontaneous pneumothorax (SP), several challenges and controversies persist in clinical practice. These issues arise from patient selection, variability in management protocols, specific population considerations and ethical or medico-legal concerns. Addressing these challenges is essential to optimizing outcomes and improving the global standard of care for spontaneous pneumothorax [54, 55].

## Identifying suitable candidates for conservative management:

The primary challenge lies in accurately identifying patients who are suitable for conservative management. Current recommendations emphasize conservative strategies for small, stable primary spontaneous pneumothorax patients. However, determining stability and predicting outcomes can be subjective:

[1] Clinical stability: Stability is commonly defined by the absence of respiratory distress, hemodynamic instability, or evidence of tension pneumothorax. However, subjective symptoms, such as mild dyspnea or discomfort, can create ambiguity in patient assessment.

[2] Pneumothorax size: Guidelines differ on the definition of "small pneumothorax." While the British Thoracic Society (BTS) uses a threshold of <2 cm measured from the lung edge to the chest wall, the American College of Chest Physicians (ACCP) often favors intervention regardless of size.

[3] Failure to standardize these criteria can result in variability in clinical decision-making, leading some patients to undergo unnecessary interventions or delays in care. Developing robust, evidence-based tools-such as predictive models integrating imaging, biomarkers and clinical scores-may address this challenge and ensure appropriate patient selection [56, 57].

## Managing pneumothorax in specific populations:

The management of spontaneous pneumothorax is more complex in certain patient populations, such as pregnant women, individuals with comorbidities and pediatric patients:

[1] Pregnant patients: Pneumothorax during pregnancy is a rare but challenging condition. Radiation exposure from chest X-rays and computed tomography (CT) scans poses risks to the developing fetus. Conservative management is preferred in clinically stable pregnant patients to avoid invasive procedures and radiation. Ultrasound serves as a safer diagnostic and monitoring tool in this population. However, close follow-up and interdisciplinary collaboration with obstetricians are essential.

[2] Patients with comorbidities: Secondary spontaneous pneumothorax, commonly seen in patients with chronic obstructive pulmonary isease, cystic fibrosis or interstitial lung disease, presents higher risks. These patients have limited pulmonary reserves and reduced pleural healing capacity, increasing the likelihood of complications such as prolonged air leaks and respiratory failure. Conservative management is less suitable in secondary spontaneous pneumothorax, as delayed intervention can lead to poor outcomes. Immediate chest tube insertion or surgical intervention is often required.

[3] Pediatric patients: In pediatric populations, conservative management is generally favored for small primary spontaneous pneumothorax cases due to their excellent recovery potential. However, long-term follow-up is necessary, as recurrence rates tend to be higher in younger individuals. Evidence remains limited in this subgroup, necessitating further research [58, 59].

## Lack of standardized definitions and protocols:

A persistent controversy in spontaneous pneumothorax management is the lack of consensus regarding key definitions, management protocols and follow-up strategies:

[1] Definitions: Terms such as "small pneumothorax" and "clinical stability" are inconsistently defined across guidelines, leading to variations in treatment decisions. Clear, globally accepted definitions are needed to harmonize clinical practice.

[2] Follow-Up protocols: While the BTS recommends serial chest X-rays to monitor conservative management, follow-up protocols are not universally standardized. Some clinicians advocate for ultrasound as a bedside, radiation-free alternative, while others prefer CT in complex cases.

[3] Ambulatory management: Guidelines vary in their recommendations for ambulatory devices such as Heimlich valves. While the European Respiratory Society (ERS) supports their use in stable patients, other organizations are more cautious due to concerns about patient compliance and complications.

[4] The lack of standardized protocols contributes to variability in care and highlights the need for unified international guidelines.

## Ethical and medico-legal considerations:

Ethical and legal challenges arise when implementing conservative management, particularly in cases where outcomes are uncertain. These include:

[1] Informed consent: Patients must be adequately informed of the risks and benefits of conservative versus invasive management. Miscommunication or incomplete discussions can lead to dissatisfaction, litigation, or poor outcomes. Ensuring that patients understand the potential for delayed intervention is essential.

[2] Risk of deterioration: Conservative management inherently carries a risk of deterioration, such as tension pneumothorax or worsening symptoms. Ethical dilemmas arise when balancing the benefits of non-invasive care against the risks of delayed treatment. Clinicians must carefully document their decision-making process to mitigate medico-legal risks.

[3] Resource availability: In resource-limited settings, conservative management may be preferred due to a lack of surgical or interventional capabilities. However, ethical concerns emerge when access to advanced care is restricted, potentially compromising outcomes.

## Future directions and research priorities:

The evolution of conservative management in spontaneous pneumothorax is promising, but several gaps in knowledge and emerging opportunities for research remain. Addressing these priorities will enhance clinical decision-making, standardize care and improve outcomes for diverse patient populations [60].

## Gaps in current evidence and unanswered questions:

Despite robust evidence supporting conservative management in primary spontaneous pneumothorax (PSP), significant gaps persist:

[1] Long-Term outcomes: While short-term studies confirm the safety and efficacy of conservative management, data on long-term outcomes, including recurrence rates, lung function and quality of life, are limited. Long-term cohort studies and randomized controlled trials (RCTs) are needed to address these gaps.

[2] Secondary spontaneous pneumothorax (SSP): Evidence for conservative management in secondary spontaneous pneumothorax remains scarce due to the higher risks of complications. Future research should explore whether stable secondary spontaneous pneumothorax patients can safely benefit from non-invasive approaches.

[3] Pediatric and elderly populations: Limited evidence exists for spontaneous pneumothorax management in pediatric and geriatric patients, who may have different risk profiles and recovery potentials.

## Innovations in diagnostic and monitoring tools:

Advancements in imaging and monitoring tools have the potential to revolutionize spontaneous pneumothorax management:

[1] Ultrasound: Point-of-care ultrasound (POCUS) is emerging as a reliable, radiation-free diagnostic tool for detecting pneumothorax. Future studies should focus on validating its role in monitoring resolution during conservative management.

[2] Wearable sensors: The development of wearable devices to monitor respiratory patterns and detect early signs of pneumothorax progression could enable real-time, remote patient monitoring.

## Potential for biomarkers to predict outcomes:

Biomarkers hold promise for identifying patients at risk of recurrence or complications. For example:

[1] Inflammatory markers: Elevated levels of biomarkers such as matrix metalloproteinases (MMPs) may predict delayed pleural healing or air leaks.

[2] Genetic markers: Screening for genetic predispositions, such as mutations in the FLCN gene (associated with Birt-Hogg-Dubé syndrome), may help stratify recurrence risk.

[3] Research focusing on integrating biomarkers into clinical practice could guide personalized treatment strategies [61, 62].

## Role of artificial intelligence in personalized pneumothorax management:

Artificial intelligence (AI) has significant potential to enhance the diagnosis, monitoring and management of SP:

[1] Predictive modeling: Machine learning algorithms can analyze large datasets to predict outcomes, recurrence risks and complications, enabling personalized treatment decisions.

[2] Imaging analysis: AI-powered tools can improve the accuracy and efficiency of pneumothorax detection on chest X-rays and CT scans, reducing diagnostic errors and supporting resource-limited settings [63].

## Conclusion:

Conservative management is a safe and effective alternative for stable primary spontaneous pneumothorax (PSP). It reduces complications, hospitalization duration, and costs while achieving comparable lung re-expansion and recurrence rates. However, defining patient selection criteria and managing complex cases like secondary spontaneous pneumothorax (SSP) remains challenging. Standardizing global guidelines and refining risk stratification are essential. Future research should focus on integrating emerging technologies and developing unified protocols for optimal patient-centered care.

## Abbreviations:

SP - Spontaneous Pneumothorax

PSP - Primary Spontaneous Pneumothorax

SSP - Secondary Spontaneous Pneumothorax

VATS - Video-Assisted Thoracoscopic Surgery

RCT - Randomized Controlled Trial

BTS - British Thoracic Society

ERS - European Respiratory Society

COPD - Chronic Obstructive Pulmonary Disease

LDH - Lactate Dehydrogenase

MMPs - Matrix Metalloproteinases

FLCN - Folliculin Gene

AI - Artificial Intelligence

POCUS - Point-of-Care Ultrasound

CT - Computed Tomography

ACCP - American College of Chest Physicians

## Funding:

This research/study/project did not receive any specific grant from funding agencies in the public, commercial, or not-for-profit sectors. All expenses related to this work were borne by the authors or their affiliated institutions.

## Figures and Tables

**Table 1:** Key differences between primary spontaneous pneumothorax and SSP

**Aspect**	**Primary Spontaneous Pneumothorax (PSP)**	**Secondary Spontaneous Pneumothorax (SSP)**
Definition	Occurs without underlying lung disease.	Occurs in patients with preexisting lung disease.
Demographics	Young, healthy individuals, mostly males.	Older individuals with lung conditions like COPD.
Etiology	Rupture of subpleural blebs.	Underlying lung pathology (*e.g.*, emphysema).
Severity	Generally mild with minimal symptoms.	Often severe due to limited pulmonary reserve.
Management	Observation or minimally invasive procedures.	Requires urgent intervention in most cases.
Recurrence	Common in untreated cases.	Higher risk due to ongoing lung disease.

**Table 2:** Key milestones in pneumothorax research and guidelines

**Year**	**Milestone**	**Impact on Management**	**Reference**
1803	Introduction of the term "pneumothorax" by Laennec	Marked the recognition of the condition as a distinct clinical entity.	Laennec, 1803
1950s	Standardization of chest tube thoracostomy	Established chest tube drainage as the primary treatment for pneumothorax.	Light, 2015
1990s	Adoption of video-assisted thoracoscopic surgery	Revolutionized surgical management with reduced morbidity and hospital stays.	MacDuff *et al.*, 2010
2000	Sahn and Heffner's pathophysiology framework	Provided a comprehensive understanding of pneumothorax pathogenesis, influencing treatment decisions.	Sahn & Heffner, 2000
2010	British Thoracic Society (BTS) guidelines	Introduced formal recommendations for conservative management of primary spontaneous pneumothorax, emphasizing observation and oxygen therapy for stable cases.	Roberts *et al.*, 2010
2020	Brown *et al.*'s study on conservative vs. interventional management of PSP	Demonstrated that conservative management is non-inferior to invasive approaches in selected cases, prompting a reevaluation of existing guidelines.	Brown *et al.*, 2020

**Table 3:** Evolution of pneumothorax management

**Era**	**Management Approach**	**Key Features**	**Advantages**	**Challenges**
Pre-Modern Era	Crude drainage techniques	Primitive methods using reeds and siphoning devices.	Provided symptom relief.	High infection risk, poor outcomes.
Early Modern Era	Chest tube drainage	Standardized placement of chest tubes for pleural air evacuation.	Reliable and effective in acute pneumothorax cases.	Painful, risk of infection, prolonged hospitalization.
Surgical Era	Thoracotomy and pleurodesis	Direct visualization and surgical intervention to prevent recurrence.	Effective in reducing recurrence rates.	Highly invasive, long recovery periods.
Minimally Invasive	video-assisted thoracoscopic surgery	Video-assisted thoracoscopy for bleb resection and pleurodesis.	Less invasive, shorter hospital stays.	Requires specialized training and facilities.
Conservative Era	Observation and oxygen therapy	Focused on non-invasive care for stable cases, relying on self-resolution.	Reduces complications, cost-effective.	Requires careful patient selection and monitoring.

**Table 4:** Key features of primary and secondary spontaneous pneumothorax

**Aspect**	**Primary Spontaneous Pneumothorax (PSP)**	**Secondary Spontaneous Pneumothorax (SSP)**
Cause	Rupture of apical blebs/bullae without underlying disease.	Air leakage from compromised alveoli due to lung disease.
Age Group	Young adults (18-40 years).	Older adults (>50 years) with comorbid conditions.
Healing	Spontaneous healing is common.	Healing may be impaired due to poor lung function.
Risk of Recurrence	Moderate, especially in smokers or untreated cases.	High due to underlying pathology.
Complications	Rare (*e.g.*, tension pneumothorax).	Frequent complications, including respiratory failure.

**Table 5:** Comparative summary of conservative vs. interventional management

**Aspect**	**Conservative Management**	**Interventional Management**
Indication	Small, stable primary spontaneous pneumothorax with minimal symptoms.	Large, unstable primary spontaneous pneumothorax or secondary spontaneous pneumothorax, persistent air leak, tension pneumothorax.
Components	Observation, oxygen therapy, analgesia.	Needle aspiration, chest tube insertion, or surgery (*e.g.*, video-assisted thoracoscopic surgery).
Imaging	Serial chest X-rays; CT if complications arise.	Imaging used pre- and post-intervention to confirm resolution.
Advantages	Non-invasive, fewer complications, lower costs, improved quality of life.	Rapid resolution, suitable for unstable cases.
Limitations	Requires close follow-up, risk of delayed complications.	Invasive, higher risk of infection, hospitalization.

**Table 6:** Comparative analysis of guidelines

**Aspect**	**BTS (2023)**	**American College of Chest Physicians (2001)**	**ERS (2023)**
Initial Approach for Small PSP	Observation or oxygen therapy.	Needle aspiration preferred.	Observation or ambulatory devices.
Management of Large PSP	Needle aspiration; chest tube if aspiration fails.	Early chest tube placement.	Needle aspiration or ambulatory devices.
SSP Management	Chest tube placement or ambulatory management.	Chest tube placement; early intervention.	Chest tube or ambulatory management.
Role of Ambulatory Devices	Recommended for stable secondary spontaneous pneumothorax and large primary spontaneous pneumothorax.	Not widely adopted.	Strongly recommended for stable cases.
Follow-Up Imaging	Serial X-rays during conservative management.	Routine imaging during follow-up.	Use of CT for recurrent or complex cases.
Pleurodesis	Recommended for recurrent primary spontaneous pneumothorax or persistent SSP.	Strongly encouraged after first recurrence.	Reserved for recurrent or secondary spontaneous pneumothorax cases.

**Table 7:** Summary of evidence from key clinical trials and meta-analyses

**Study**	**Design**	**Interventions**	**Key Findings**
Brown *et al.* (2020)	Multicenter randomized controlled trials	Conservative vs. invasive	Conservative management is non-inferior to invasive treatments, with fewer complications and higher satisfaction.
Lee *et al.* (2020)	Systematic review/meta-analysis	Chest tube vs. conservative	No significant difference in recurrence rates; fewer complications and shorter hospital stays with conservative management.
Hung *et al.* (2021)	Meta-analysis	Conservative vs. surgical	Conservative management reduces complications and hospital stays but has higher recurrence rates.
Lichtenstein *et al.* (2005)	Prospective study	Ultrasound for spontaneous pneumothorax diagnosis	Point-of-care ultrasound (POCUS) demonstrated high sensitivity and specificity for pneumothorax diagnosis, enabling radiation-free bedside management.
Ashby *et al.* (2014)	Cochrane review	Conservative vs. invasive	Observation and oxygen therapy are effective and safe for stable PSP; more high-quality trials are needed.
Chambers *et al.* (2019)	Meta-analysis	Ambulatory vs. invasive devices	Ambulatory devices reduce hospital stays, improve patient satisfaction and do not increase complication rates.
Al-Shamiri *et al.* (2020)	Network meta-analysis	Various interventions	Less invasive approaches, such as needle aspiration, are effective and associated with fewer complications.

**Table 8:** Comparative outcomes of conservative vs. invasive management

**Outcome**	**Conservative Management**	**Invasive Management**
Recurrence Rates	Comparable for PSP; higher for SSP.	Lower for SSP; significantly reduced with pleurodesis.
Complications	Lower risk of infection, bleeding and air leaks.	Higher risk of procedural complications.
Mortality	Rare for PSP; depends on underlying disease in SSP.	Rare for PSP; procedural risks in SSP.
Hospitalization Duration	Minimal or none (outpatient care).	Prolonged for chest tube or surgical recovery.
Cost-Effectiveness	Lower costs; avoids procedural expenses.	Higher costs due to hospital stays and procedures.
Quality of Life	Higher short-term quality of life; reduced disruption.	Lower short-term due to procedural impact; better long-term after surgery.
Subgroup-Specific	Highly effective in small, stable primary spontaneous pneumothorax.	Necessary for larger primary spontaneous pneumothorax, secondary spontaneous pneumothorax, or recurrent cases.
